# Regulation of translation in response to iron deficiency in human cells

**DOI:** 10.1038/s41598-024-59003-9

**Published:** 2024-04-11

**Authors:** Mireia S. Puig-Segui, Carolyn J. Decker, Hanna Barlit, Vyacheslav M. Labunskyy, Roy Parker, Sergi Puig

**Affiliations:** 1 Departamento de Biotecnología, Instituto de Agroquímica y Tecnología de Alimentos (IATA), Consejo Superior de Investigaciones Científicas (CSIC), Calle Catedrático Agustín Escardino 7, 46980 Paterna, Valencia Spain; 2 Department of Biochemistry, University of Colorado Boulder, Boulder, CO USA; 3 Escuela Técnica Superior de Ingeniería Agronómica y del Medio Natural (ETSIAMN), Universidad Politécnica de Valencia (UPV), Valencia, Spain; 4 Howard Hughes Medical Institute, University of Colorado Boulder, Boulder, CO USA; 5 Department of Dermatology, Boston University School of Medicine, Boston, MA 02118 USA; 6 BioFrontiers Institute, University of Colorado Boulder, Boulder, CO USA

**Keywords:** Autophagy, Cell death, Cellular imaging, Post-translational modifications, Molecular biology, Post-translational modifications, Translation, Cell biology, Cell growth, TOR signalling

## Abstract

Protein synthesis is a highly energy-consuming process that is downregulated in response to many environmental stresses or adverse conditions. Studies in the yeast *Saccharomyces cerevisiae* have shown that bulk translation is inhibited during adaptation to iron deficiency, which is consistent with its requirement for ribosome biogenesis and recycling. Although iron deficiency anemia is the most common human nutritional disorder, how iron modulates translation in mammals is poorly understood. Studies during erythropoiesis have shown that iron bioavailability is coordinated with globin synthesis via bulk translation regulation. However, little is known about the control of translation during iron limitation in other tissues. Here, we investigated how iron depletion affects protein synthesis in human osteosarcoma U-2 OS cells. By adding an extracellular iron chelator, we observed that iron deficiency limits cell proliferation, induces autophagy, and decreases the global rate of protein synthesis. Analysis of specific molecular markers indicates that the inhibition of bulk translation upon iron limitation occurs through the eukaryotic initiation factor eIF2α and mechanistic target of rapamycin (mTOR) pathways. In contrast to other environmental and nutritional stresses, iron depletion does not trigger the assembly of messenger ribonucleoprotein stress granules, which typically form upon polysome disassembly.

## Introduction

Protein synthesis is a highly energy-consuming cellular process. Therefore, in response to adverse conditions, such as oxidative stress, osmotic shock, or nutritional deprivation of glucose or amino acids, eukaryotic cells inhibit translation^1^. The rate-limiting stage in translation is initiation, which is subject to multiple regulations^2^. An important pathway regulating translation is mechanistic target of rapamycin (mTOR), which limits overall protein synthesis in response to unfavorable environmental cues^3^. Under optimal conditions, mTOR complex 1 (mTORC1) kinase phosphorylates the eIF4E-binding protein (4EBP1) and disrupts its association with the cap-binding factor eIF4E, leading to efficient translation. Upon stress, mTOR is inactivated, leading to 4EBP1 dephosphorylation, which favors binding to eIF4E and inhibition of global translation^4^. Another central pathway regulating protein synthesis involves the translation initiation factor eIF2. Eukaryotic translation is initiated by the assembly of the ternary complex consisting of eIF2, GTP, and the initiator methionyl-tRNA (tRNA^Met^). Upon stress, the alpha subunit of the eIF2 factor (eIF2α) is phosphorylated, leading to the inactivation of the ternary complex in a GDP-bound form and the repression of bulk translation^2^.

In mammals, multiple stresses including glucose deprivation, severe heat shock, azide (N_3_^−^) and arsenite (AsO_2_^−^) treatments induce eIF2 phosphorylation and translation repression, which leads to the formation of membrane-less ribonucleoprotein (RNP) condensates called stress granules (SGs)^5,6^. SGs assemble through a stepwise process that occurs through a combination of specific protein–protein, protein-RNA, and RNA-RNA interactions. The RNA-binding protein G3BP1, and its paralog G3BP2, form the central node for the condensation of RNPs into SGs^7,8^. SGs are dynamic RNPs composed of mRNAs stalled at translation initiation, translation initiation factors, small but not large ribosomal subunits, poly(A)-binding protein (PABP1), tRNA synthetases, RNA-binding proteins, proteins with prion-like motifs, and multiple ATPases^9^, although their exact composition can vary under different conditions. SGs have attracted considerable attention due to their critical roles in neurodegenerative diseases, viral infections, cancer, and stress responses, although their physiological roles are not fully understood^6,10^.

Iron is an essential element for all eukaryotic organisms because it is involved as a redox cofactor in many cellular processes, including oxygen transport, energy generation via mitochondrial respiration, and DNA, protein and lipid biosynthesis. The low solubility of ferric iron at physiological pH has made iron deficiency anemia into one of the most common nutritional disorders worldwide^11^. In response to iron starvation, mammals upregulate iron acquisition, distribution, and mobilization from reservoirs. Iron metabolism is regulated at the cellular level by the iron regulatory protein/iron responsive element (IRP/IRE) system and at the systemic level through the hormone hepcidin^12^. In response to iron deficiency, proteins IRP1 and IRP2 bind to IREs within the 5ʹ untranslated region (UTR) of specific mRNAs, including ferritin H and L (*FTH1* and *FTL1*), ferroportin (*FPN1*), and hypoxia inducible factor 2α (*HIF2α*), blocking their translation^13^. Conversely, IRP binding to the 3ʹUTR of transferrin receptor 1 (*TfR1*) and divalent metal transporter 1 (*DMT1*) increases their mRNA stability^14^. TfR1 is a transmembrane protein that interacts with transferrin and mediates iron-bound transferrin acquisition to many tissues^12^. Tristetraprolin (TTP), a protein primarily involved in anti-inflammatory responses, promotes the downregulation of non-essential iron-dependent processes^15^.

Iron is required for protein synthesis by a conserved and indispensable iron-sulfur-containing protein, designated ABCE1 in mammals and Rli1 in the yeast *Saccharomyces cerevisiae*, which participates in ribosome biogenesis and recycling^16–18^. Additionally, several steps of translation depend on iron-containing enzymes required for specific modifications of translation factors and transfer RNAs^19^. By using *S. cerevisiae* as a model, we have shown that iron depletion limits bulk translation via both mTORC1 and eIF2α phosphorylation^20,21^. The regulation of protein synthesis by iron has been extensively studied during mammalian erythropoiesis^22^. The main function of erythrocytes is to assemble globins and heme into hemoglobin for oxygen transport to tissues. Thus, during heme deficiency, the heme-regulated inhibitor HRI phosphorylates eIF2α and inhibits mTORC1 signaling to limit protein synthesis in order to coordinate iron and globin α and β chains availability in red blood cells^23,24^. Suppression of mTORC1 signaling has also been reported in iron-depleted intestinal Caco-2 cells^25^. However, it remains unstudied how iron regulates translation in other mammalian cell types.

In this report, we utilized osteosarcoma U-2 OS cells, a well-acknowledged model for studying the dynamics of messenger RNP assembly^26^, to investigate the molecular and cellular mechanisms that human cells use to control translation during iron restriction.

## Results

### Iron deficiency limits cell proliferation and activates autophagy

**Figure 1:**
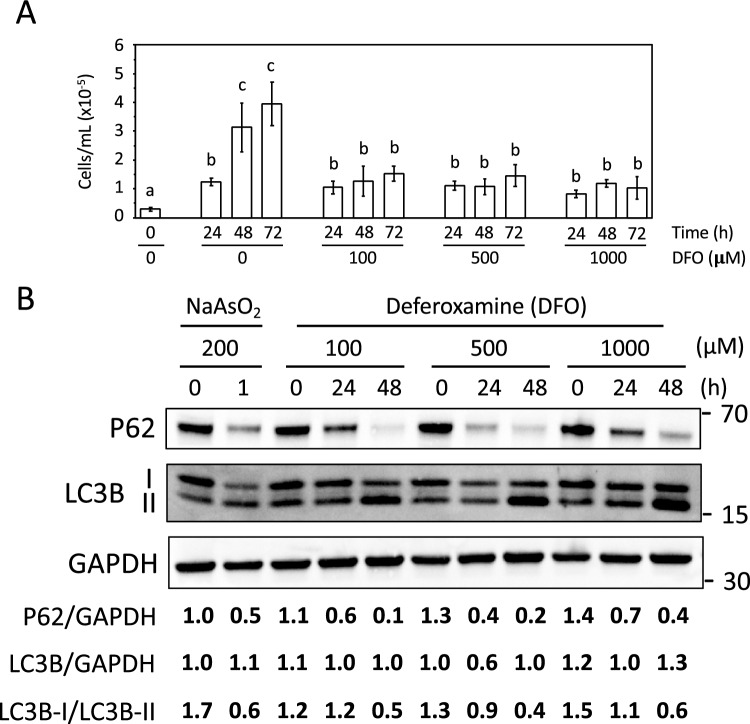
Limitation of iron bioavailability in U-2 OS cell cultures arrests cell growth and promotes autophagy. U-2 OS cells were cultured for 3 days in the presence of the indicated concentrations of NaAsO_2_ or DFO. (**A**) Cell number was determined at the indicated time points. At least 3 independent biological replicates were performed, and the mean and standard deviation were calculated and represented. Different letters above bars indicate statistically significant differences (p-value < 0.02). (**B**) Total proteins were extracted, and P62, LC3B and GAPDH protein levels were determined by immunoblotting with specific antibodies. P62 and LC3B protein levels were expressed relative to those of GAPDH in order to normalize the results. The ratio LC3B-I/ LC3B-II is also indicated. The molecular weight (kDa) is represented on the right. A representative experiment of two independent biological replicates is shown.

Since iron is essential for any eukaryotic organism, we examined whether the addition of the Fe^3+^-chelator deferoxamine (DFO) to culture media affects human cell growth. We began by inoculating the human osteosarcoma U-2 OS cell line at 30,000 cells/mL (confluence < 10%) into media containing increasing concentrations of DFO (0, 100 μM, 500 μM, and 1 mM). Cells were imaged and counted every 24 h for three consecutive days using a cell counter (“Methods”). It was observed that, under normal conditions (without DFO addition), the U-2 OS culture grew from 3.0 × 10^4^ to 4.0 × 10^5^ cells/mL during the 3-day incubation period (Fig. 1; 0 μM DFO). DFO did not affect the growth of the culture during the first 24 h. However, cell proliferation was significantly reduced during the next two days of iron limitation (Fig. 1, 100–1000 μM DFO). There were no significant differences between the different concentrations of DFO used (Fig. 1), indicating that 100 μM DFO effectively chelated all available extracellular iron. In addition, consistent with previous research demonstrating G1/S arrest in response to iron depletion^27^, we observed a notable increase in cell size as the bioavailability of environmental iron decreased (Fig. S1). These results suggest that iron is critical for cell growth and division beyond 24 h.

To further evaluate the physiological status of iron-deprived U-2 OS cells, we assessed cell viability by quantifying the number of dead cells in the population. To accomplish this, we used a luminescence assay that evaluates the protease activity released from dead cells that have lost membrane integrity (see “Methods”). Cell viability was greater than 75% during the first three days of iron depletion, with no significant differences compared to iron replete conditions.

We also investigated the effect of iron depletion on autophagy, which is a pro-survival catabolic process activated during stress to recycle damaged components and nutrients^28^. We used two different markers, namely P62 and microtubule-associated proteins 1A/1B light chain 3B (LC3) proteins^29^, to evaluate whether iron depletion promotes autophagy in U-2 OS cells. P62 is an autophagy receptor whose protein levels decrease during autophagy. LC3B protein exists in a cytosolic state (LC3B-I) under normal conditions, but it becomes lipidated and recruited to the autophagosome membrane during autophagy. The lipidated LC3B-II form can be distinguished from the soluble LC3B-I form because of its faster electrophoretic mobility on SDS-PAGE, ~ 16 kDa compared to ~ 18 kDa, respectively^30^. We used sodium arsenite treatment as a positive control because it promotes autophagy, as indicated by the drop in P62 protein levels and the shift from the LC3B-I to the LC3B-II form (Fig. 1B).

Finally, we observed that iron deficiency led to autophagy activation as assessed by both a decrease in P62 levels, and an increase in the abundance of the faster migrating LC3B-II form (Fig. 1B). These findings suggest that iron deficiency promotes autophagy, which may enhance survival under these conditions.

### Transferrin receptor and tristetraprolin levels increase in response to iron deficiency

**Figure 2:**
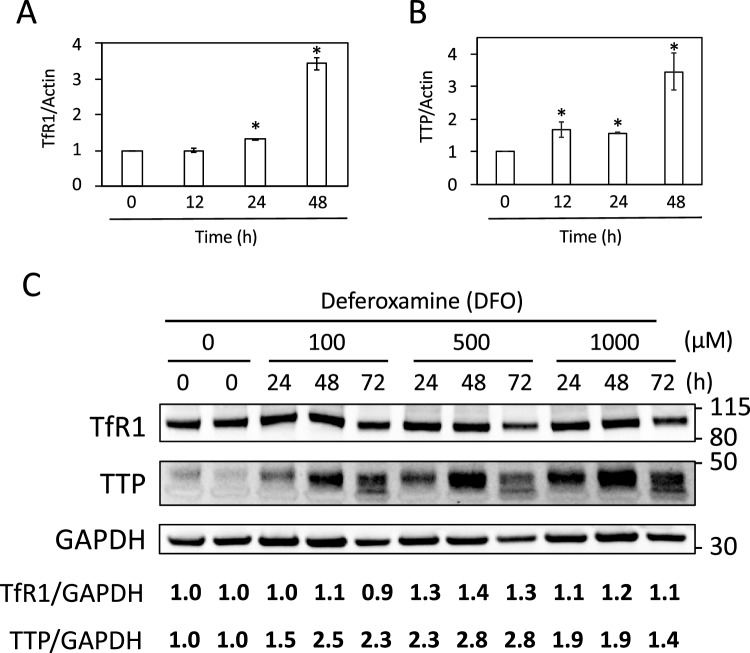
Induction of transferrin receptor and tristetraprolin expression upon iron limitation. (**A** and **B**) U-2 OS cells were treated with 100 µM DFO for 12, 24, and 48 h. RNA was extracted and the mRNA expression of TfR1 (**A**) and TTP (**B**) was analyzed by RT-qPCR. Graphs show the mean and standard deviation of three independent experiments. Statistical analysis compares samples with time zero. An asterisk above the bars indicates statistically significant differences (p < 0.05). (**C**) U-2 OS cells were cultured for 3 days in the presence of the indicated concentrations of DFO, and proteins were extracted at the indicated time points and analyzed by immunoblotting (see “Methods” section for details). Antibodies specific for TfR1, TTP and GAPDH were used. Glyceraldehyde-3-phosphate dehydrogenase (GAPDH) protein levels were used as loading control. TfR1 and TTP protein levels were expressed relative to those of GAPDH in order to normalize the results. The molecular weight (kDa) is shown on the right. A representative experiment of two independent biological replicates is shown.

Both TfR1 and TTP are iron biomarkers whose expression has been shown to increase in response to iron deficiency conditions^15^. Therefore, we assessed whether TfR1 and TTP expression were induced in response to DFO treatment to ensure that U-2 OS cells were sensing iron deficiency. For this purpose, U-2 OS cells were cultured in increasing concentrations of DFO, and then RNA and proteins were extracted for further analysis (see “Methods” section for details). Consistent with iron limitation, the mRNA levels of both TfR1 and TTP increased after 48 h of incubation (Fig. 2A and B). Although TfR1 mRNA levels increased rapidly, only a small increase in protein levels was observed during adaptation to iron deficiency (Fig. 2C). A clear induction of TTP was observed during the first two days of iron depletion, whereas no further induction was obtained on the third day (Fig. 2C). Only slight differences were observed between the different DFO concentrations used (Fig. 2C). Thus, these biomarkers confirm that DFO limits the bioavailability of iron in U-2 OS cells.

### Translation is repressed in response to iron deficiency

**Figure 3:**
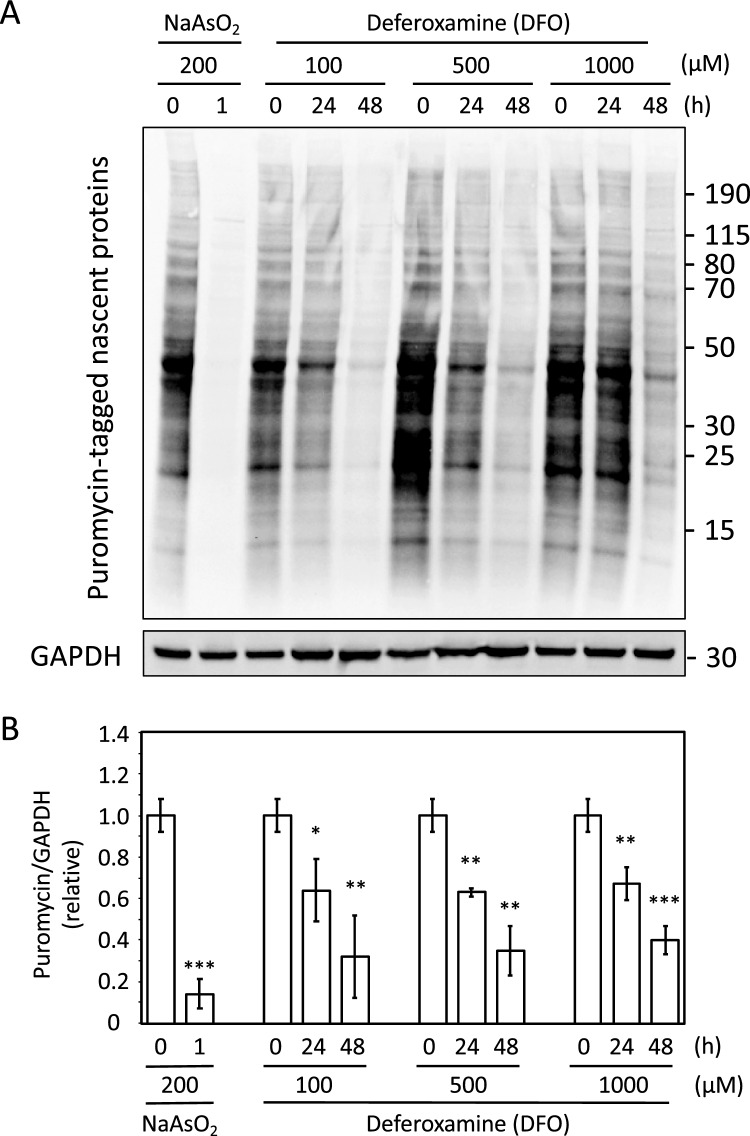
Translation is repressed during iron deficiency. U-2 OS cells were cultured for 2 days in the presence of the indicated concentrations of DFO, and treated with a 5-min pulse of puromycin at the indicated time points. Treatment with 200 μM sodium arsenite (NaAsO_2_) for 1 h was used as a control^32^. Proteins were extracted and analyzed by immunoblotting (see “Methods” section for details). Antibodies specific for puromycin and GAPDH were used. The Puromycin/GAPDH ratio is represented. The molecular weight (kDa) is shown on the right. (**A**) A representative experiment is shown. (**B**) The mean and standard deviation of 3 independent biological experiments are represented. Statistical analysis compares samples with time zero. Asterisks above the bars indicate statistically significant differences (*p < 0.05; **p < 0.01; and ***p < 0.001).

We then examined the impact of iron deficiency on translation. For these experiments we used puromycin, which is a translation inhibitor because it mimics a tyrosyl-tRNA that causes the labeling and release of elongating polypeptide chains from translating ribosomes. Here, we used a 5-min pulse of 10 mg/L of puromycin and determined its incorporation into nascent peptides as a measure of bulk translation^31^. For this purpose, U-2 OS cells were treated with increasing concentrations of DFO (0, 100, 500 and 1000 μM) and cells were harvested at 0, 24 and 48 h after the 5 min pulse of puromycin. As a positive control, U-2 OS cells were treated for 1 h with 200 μM sodium arsenite (NaAsO_2_), which induces oxidative stress that stops bulk translation^32^. An immunoblot with anti-puromycin antibody (α-puro) was used to detect puromycin levels in all protein lysates (Fig. 3A). GAPDH was used as a loading control to normalize puromycin levels (Fig. 3A). Three biological replicates were performed, and puromycin/GAPDH values were plotted and related to each corresponding time zero (Fig. 3B). As expected, arsenite treatment strongly decreased puromycin incorporation (Fig. 3, ~ 15% of the initial values). Importantly, we observed that DFO decreases puromycin incorporation to ~ 65% of the initial rates after 24 h of treatment and to 35–40% after 48 h (Fig. 3). No significant differences were observed between the different concentrations of DFO. From these results, it can be concluded that cells under iron-deficient conditions progressively decreases bulk translation, although not as strongly as arsenite treatment.

### eIF2α is phosphorylated in response to iron-deficient conditions

**Figure 4:**
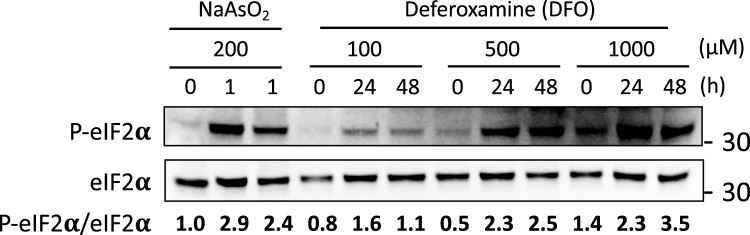
eIF2α is phosphorylated in response to iron deficiency. U-2 OS cells were cultured as described in Fig. 3. Proteins were extracted and analyzed by immunoblotting (see “Methods” section for details). Antibodies specific for total eIF2α and phosphorylated eIF2α (P-eIF2α) were used. The ratio of P-eIF2α/eIF2α is shown. Molecular weight size (kDa) is indicated on the right. A representative experiment of different independent biological replicates is shown.

Upon addition of arsenite, eukaryotic translation initiation factor elF2α is phosphorylated, resulting in translation repression^32^. Therefore, we investigated whether eIF2α was phosphorylated upon DFO addition. For this purpose, U-2 OS cells were cultured with increasing concentrations of DFO, and proteins were extracted every 24 h for three days (see “Methods” section for details). As a positive control, U-2 OS cells were treated with 200 μM arsenite for one hour. Levels of phosphorylated eIF2α (P-eIF2α) were assessed by immunoblotting with an antibody that recognizes only its phosphorylated form. These values were normalized to total eIF2α by using an antibody that recognizes both phosphorylated and non-phosphorylated eIF2α, and were made relative to time zero (Fig. 4). The P-eIF2α/eIF2α ratio indicated a ~ threefold increase in eIF2α phosphorylation after an hour of incubation with arsenite (Fig. 4). Treatment with DFO also promoted eIF2α phosphorylation, with higher P-eIF2α/eIF2α levels achieved when high DFO concentrations were used (Fig. 4). From these results, we conclude that iron deficiency leads to the phosphorylation of eIF2α in U-2 OS cells.

### eIF4E-binding protein is dephosphorylated in response to iron-deficient conditions

**Figure 5:**
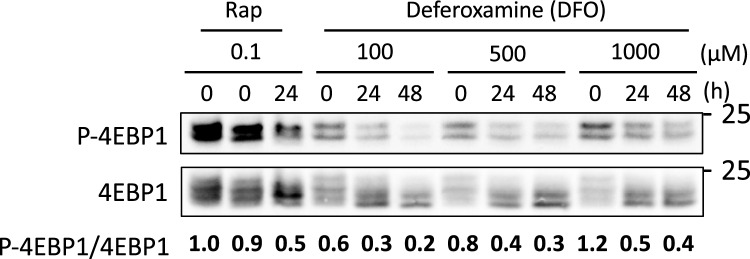
4EBP1 is dephosphorylated in response to iron deficiency. U-2 OS cells were cultured and proteins were extracted as described in Fig. 4. Specific antibodies for total 4EBP1 and phosphorylated 4EBP1 (P-4EBP1) were used. The P-4EBP1/4EBP1 ratio is represented. The molecular weight (kDa) is indicated on the right. A representative experiment of two independent biological replicates is shown.

Mammalian target of rapamycin (mTOR) limits overall protein synthesis in response to adverse environmental cues^3^. To determine whether mTOR modulates bulk translation during iron deficiency, we determined the phosphorylation state of the eIF4E-binding protein (4EBP1), which decreases under stress conditions due to a drop in mTOR activity, resulting in recruitment of eIF4E and translation impairment^3^. U-2 OS cells treated with 100 nM rapamycin, a specific mTOR inhibitor, for 24 h were used as a control. U-2 OS cells were cultured at different concentrations of DFO, and the phosphorylation state of 4EBP1 was determined by immunoblotting with an antibody that recognizes only its phosphorylated form. Values were normalized to total 4EBP1 using an antibody that recognizes both phosphorylated and non-phosphorylated 4EBP1, and made relative to time zero (Fig. 5). The P-4EBP1/4EBP1 ratio indicated a drop in 4EBP1 phosphorylation after both rapamycin and iron depletion (Fig. 5). These results indicate that iron deficiency leads to the dephosphorylation of 4EBP1 in U-2 OS cells.

Taken together, our results suggest that iron deficiency can lead to phosphorylation of eIF2α and the inhibition of mTOR, leading the dephosphorylation of 4EBP1 and subsequent inhibition of eIF4E and its role in translation initiation. This provides two distinct molecular mechanisms by which iron deficiency reduces translation.

### Iron depletion does not activate the assembly of stress granules

**Figure 6:**
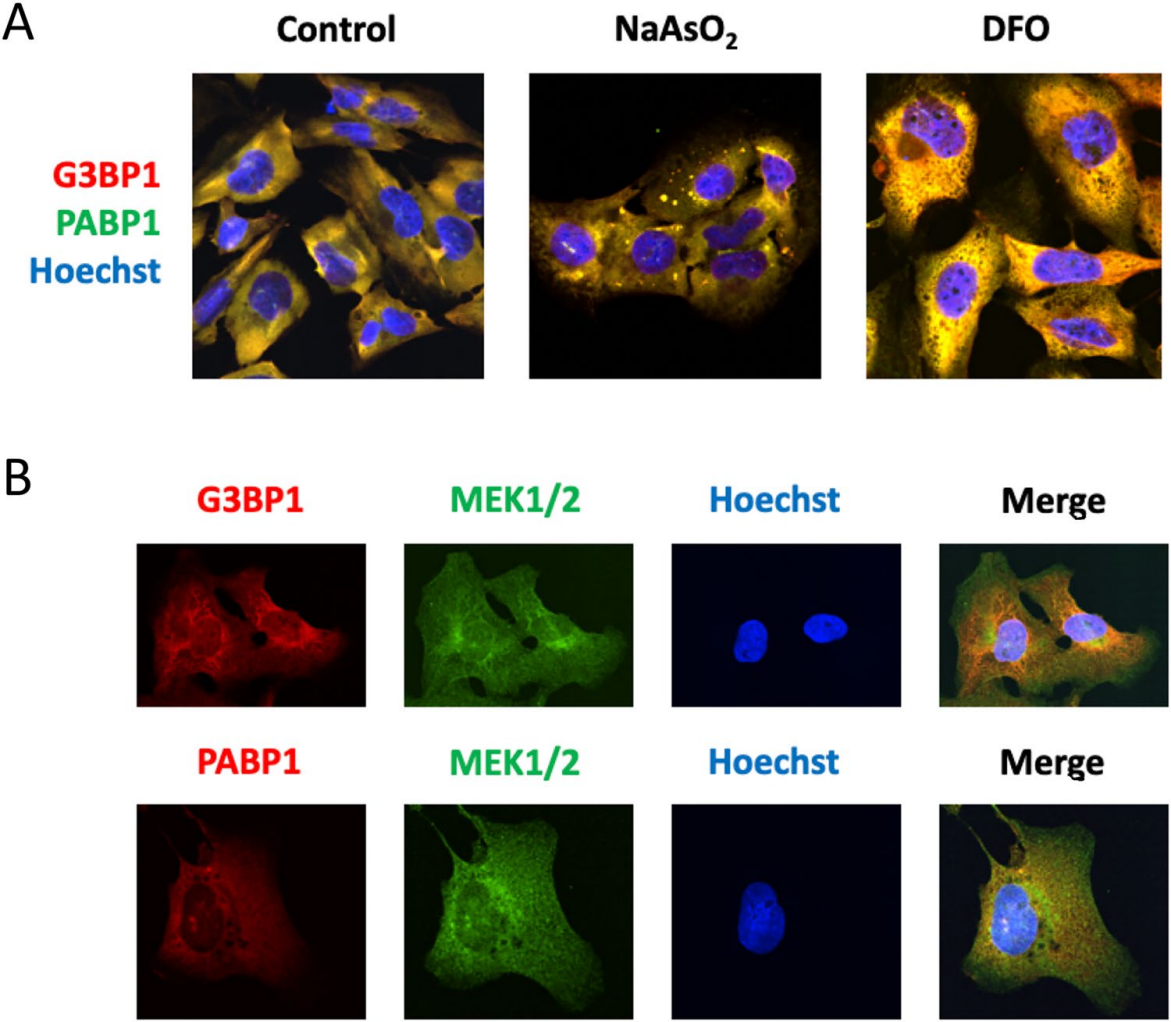
Iron deficiency does not promote stress granule assembly. (**A**) U-2 OS cells were cultured in the presence of NaAsO_2_ (1 h, 200 μM) or DFO (500 μM or 1 mM). DFO samples were analyzed at 12 h, 24 h 48 h and 72 h with similar results. Images from 2 days at 500 μM DFO are shown. (**B**) SG components are cytosolic under iron-depleted conditions. Cells were cultured in 500 μM DFO for 2 days and the subcellular localization of G3BP1, PABP and MEK1/2 was determined by immunofluorescence.

In response to stresses that limit translation initiation and lead to ribosomes running off mRNAs, mammalian cells activate the assembly of SGs. To address whether iron limitation promotes the formation of SGs, we determined the subcellular localization of two SG-specific components during iron deficiency, G3BP1 and PABP1, by immunofluorescence using specific antibodies. Under normal growth conditions, both proteins were diffusely localized, whereas they accumulated in cytosolic foci (SGs) upon treatment with sodium arsenite (Fig. 6A). Remarkably, iron depletion did not promote the assembly of either G3BP1 or PABP1 containing granules in U-2 OS cells (Fig. 6A, control and NaAsO_2_). Instead, a distinct overlapping localization pattern was observed for both proteins (Fig. 6A, DFO). To ascertain whether this localization pattern was consistent with cytosolic localization, we colocalized both G3BP1 and PABP1 proteins with MEK1 and MEK2 proteins, which are known to be cytosolic^33^ (Fig. 6B). These results suggest that iron deficiency does not promote the assembly of SGs in U-2 OS cells, as G3BP1 and PABP1 proteins do not accumulate in foci, but display a diffuse pattern consistent with cytosolic localization.

## Discussion

Iron is an essential micronutrient for all eukaryotic organisms and its depletion in mammalian cells causes cell cycle arrest at G1/S^27^. Consistent with this, we have observed that iron deficiency limits U-2 OS cell growth. In yeast, iron depletion also arrests growth, but cells are viable during long periods of iron limitation^34^. Here, we observed that U-2 OS cell viability did not decrease after 3 days of iron starvation, despite growth arrest. One process that could contribute to cell survival under low iron conditions is autophagy, which recycles cellular components and nutrients^28^. Iron depletion has been suggested to promote autophagy in *S. cerevisiae* and in specific human cell types such as pancreatic cancer, myeloma, and Parkinson’s disease model cells, probably due to the inactivation of the mTORC1 pathway^20,34–39^. In this study, we observed a decrease in p62 and the accumulation of LC3B-II after 24 h of iron depletion, which is consistent with U-2 OS cells activating autophagy. However, a recent study has shown that iron-deficient hepatocytes accumulate both p62 and LC3B-II markers, and assays with autophagy inhibitors suggest a decreased autophagic flux^40^. Therefore, the modulation of autophagy during iron deficiency and its physiological implications in different tissues and specific pathogenic conditions require further investigation^41^.

As expected for an iron-dependent process, we observed a significant decrease in bulk translation in response to iron deficiency. Similar to previous studies in yeast and erythroid cells^20,23,24,42,43^, we show that U-2 OS cells limit global translation by phosphorylating eIF2α and inhibiting mTORC1 signaling when iron is scarce. In yeast, Gcn2 kinase limits protein synthesis during iron deficiency by phosphorylating eIF2α through a mechanism dependent on the uncharged tRNA-sensing Gcn1-Gcn20 complex^21^. In red blood cells, HRI kinase senses low heme levels and phosphorylates eIF2α, thereby inhibiting globin synthesis^23^. Although mammalian cells possess four eIF2α kinases, little is known about the kinases responsible for eIF2α phosphorylation upon iron deficiency in other tissues. We have recently shown that iron depletion also leads to the inhibition of mTORC1 signaling through a mechanism conserved from yeast to humans^39^. The downregulation of mTOR signaling has also been observed in Caco-2 cells from human intestinal epithelium, COS-1 fibroblast-like cells from monkey kidney, and rats deficient in iron^25,44^. Crosstalk between both pathways has been reported in both yeast and mammals. Studies during erythropoiesis indicate that HRI kinase suppresses mTORC1 signaling during iron deficiency^24^. In yeast, inhibition of mTOR provokes the dephosphorylation and activation of Gcn2 through the action of Sit4 phosphatase^45^. Similarly, phosphorylation of eIF2α by mTOR inhibition is necessary for the activation of autophagy in mammalian cell lines^46^. In addition to mechanisms that regulate bulk translation, eukaryotic cells express mRNA-binding proteins, such as IRPs in mammals and Cth2 in yeast, that repress the translation of multiple mRNAs implicated in iron metabolism upon iron starvation^13,47,48^. The contribution of this set of transcripts to the global repression of translation in response to iron deficiency is currently unknown.

Eukaryotic cells form SGs in response to a variety of environmental cues including oxidative stress (e.g., arsenite), heat shock, osmotic stress, viral infection, and UV irradiation^6^. However, no clear SG assembly was observed when U-2 OS cells were depleted of iron. Different reasons could explain this observation. Stress and other mRNP granules are formed by multivalent RNA-RNA, RNA–protein, and protein–protein interactions that occur in separable assembly stages and range from smaller condensates to microscopically visible foci depending on cell types and the severity of the stress^49,50^. Previous studies in yeast showed that iron deficiency leads to an important decrease in total and messenger RNA due to an mTORC1-dependent downregulation of the activity of all RNA polymerases^20^. Therefore, a possibility is that SG foci do not form during iron deficiency in mammalian cells due to a decrease in bulk RNA levels. For example, activation of ribonuclease L in response to double-stranded RNAs represses translation by promoting a widespread turnover of mRNAs that limits SG formation^51^. Another possibility is that the degree of translation repression during iron deficiency is not sufficient to trigger SG formation. Compared to arsenite (~ 90% repression), translation is more modestly repressed in response to iron starvation (~ 60–65% repression), highlighting the difference between an acute stress and adaptation to nutritional deprivation. Finally, other stresses that repress translation, such as prolonged nutrient starvation and energy deficiency, lead to the formation of smaller assemblies denoted starvation-induced and energy-deficiency induced SGs, respectively, which differ in composition and function from canonical SGs^52,53^. Therefore, although these membrane-less assemblies were not visible microscopically, we cannot exclude the formation of small non-canonical aggregates or the contribution of SG components such as G3BP to the regulation of translation during adaptation to iron limitation.

## Methods

### Cell culture and drug treatments

Human osteosarcoma U-2 OS cells (obtained from the American Type Culture Collection, ATCC) were cultured in an incubator under standard conditions at 37 °C with 5% CO_2_ to promote growth and maintenance. Cultures were expanded in T-25 or T-75 flasks containing 5 and 15 mL, respectively, of sterile DMEM medium (homemade and stored refrigerated) supplemented with 10% fetal bovine serum (FBS) and 1% penicillin–streptomycin, and allowed to adhere overnight. Cells were split under sterile conditions (the chamber was previously cleaned with 70% ethanol), at least every 3 days to maintain them below 80% confluence. Cell confluency is defined as the proportion of the culture dish or flask that is covered by adherent cells. Briefly, the medium was aspirated and cells were washed with one volume of Dulbecco′s phosphate buffered saline (DPBS) at room temperature. Cells were treated with 0.1 volume of 1X trypsin (thawed from the freezer) for at least 1 min at 37 °C. This facilitated protein digestion and allowed the cells to be suspended in a round shape. We then diluted the cells in fresh medium and a new flask. Deferoxamine (DFO, Sigma) was added at 100, 500 and 1000 μM to induce iron deprivation. No differences were observed with the different concentrations of DFO used. Treatments with 200 μM NaAsO_2_ (Sigma) or 1 nM rapamycin served as controls.

### Cell counting and viability assays

Cells were seeded on 24-well plates at an initial concentration of 3 × 10^4^ cells/mL. After overnight cell attachment, DFO was added at the indicated final concentration. An SD100 cell counter (Nexcellom Bioscience) was used to quantify cell number. To determine viability, we followed the manufacturer's instructions for the CytoTox-Glo™ cytotoxicity assay (Promega).

### Cell harvesting and protein extraction

Cells were seeded on 6-well plates (protein extraction) at different initial concentrations to avoid confluence during the harvesting process. We used 100,000 cells/mL for time 0, 50,000 cells/mL for 24 h, and 25,000 cells/mL for 48 and 72 h. U-2 OS cultures were also treated with DFO at the indicated concentrations for iron depletion.

To harvest cells for protein extraction, medium was aspirated and cells were washed with one volume of cold DPBS. The DPBS was removed and 125 μL of cold lysis buffer (150 mM NaCl, 50 mM Tris–HCl pH 7.4, 1 mM EDTA, 1% (v/v) Triton-X100, 1× of protease inhibitor (one tablet of Complete Mini, EDTA-free protease inhibitor cocktail (Roche) and another tablet of PhosSTOP Easypack protease inhibitor cocktail tablets (Roche) in 10 mL) and fresh 1 mM DTT were added. Cells were scraped from the plates with a scraper and transferred to Eppendorf tubes, which were immediately placed on ice. The cells were then vortexed every few minutes for 20 min with the cells on ice in between. Finally, the samples were centrifuged at 13,000*g* for 10 min at 4 °C to separate the cell debris (pellet). Proteins were transferred from the supernatant to a new Eppendorf tube, frozen in liquid nitrogen, and stored at − 20 °C for storage.

### RNA extraction and analysis

10^5^ U-2 OS cells were seeded into each well of the 6-well plates. After overnight incubation, cells were treated with 100 µM DFO for 12 and 24 h. Cells were washed with 5 mL of cold PBS, and total RNA was isolated using Direct-zol RNA Miniprep kit (Zymosearch). RNA was treated with DNase I, and 2 μg of RNA was used for cDNA synthesis using SuperScript III reverse transcriptase (Thermo Fisher Scientific) with random hexamer primers according to manufacturer’s instructions. mRNA expression of TfR1 and TTP were then analyzed by real-time PCR using KAPA SYBR FAST qPCR Master Mix (Kapa Biosystems) and the CFX-96 Touch Real-Time PCR Detection System (Bio-Rad Laboratories). β-actin was used as a reference gene for normalization of mRNA expression between genotypes. The following primer pairs were used: TfR1: 5ʹ-GGTCAAAGACAGCGCTCAAAA-3ʹ and 5ʹ-CAACCTTTTCTGCAAAGGTG-3ʹ; TTP: 5ʹ-CGCTACAAGACTGAGCTAT-3ʹ and GAGGTAGAACTTGTGACAGA; β-actin: 5ʹ-TTTTGGCTATACCCTACTGGCA-3ʹ and 5ʹ-CTGCACAGTCGTCAGCATATC-3ʹ.

### Protein quantification, electrophoresis, and immunoblotting

First, samples were thawed on ice and their protein concentration was determined by using the Qubit Broad Protein Assay (Invitrogen) and an iQubit Fluorometer (Invitrogen). Between 30 and 60 μg of total protein were mixed with 4X SDS loading buffer (200 mM Tris-HCI pH 6.8, 8% SDS, 0.4% bromophenol blue and 40% glycerol) to obtain a final 1X concentration. Samples were then heated at 95 °C for 5 min and loaded onto pre-cast NuPAGE 4–12% Bis–Tris gels (Invitrogen) for denaturing electrophoresis. Upon completion of electrophoresis, proteins were transferred onto a nitrocellulose membrane using iBlot 2 NC stacks (Invitrogen) and iBlot 2 (Invitrogen). Ponceau staining was performed to verify proper protein transfer and loading. Membranes were washed three times with 1X TBS-T and blocked with 5% bovine serum albumin (BSA, Merck) in 1X TBS with 0.1% Tween 20 (1X TBS-T) for at least 1 h at room temperature with shaking. The membrane was then incubated with primary antibodies at recommended dilutions in 1X TBS-T with 5% BSA, and incubated overnight at 4 °C with shaking. Primary antibodies included rabbit α-P62 (1:1000), rabbit α-LC3B (1:1000, NB600-1384, Novus Biologicals), mouse α-GAPDH (1:1000, clone 0411, sc-47724 HRP, Santa Cruz Biotech), mouse α-TfR1 (1:500, 13–6800, Thermo Fischer Scientific), rabbit α-TTP (1:1000, clone D1I3T, 9721S, Cell Signaling Tech), mouse α-puromycin (1:10,000, clone 12D10, MABE343; Sigma-Aldrich), rabbit α-eIF2α (1:2000, 9722S, Cell Signaling Tech), rabbit α-phosphorylated eIF2α (1:2000, 9721S, Cell Signaling Tech), rabbit α-4EBP1 (1:2000, clone 53H11, 9644S, Cell Signaling Tech), and rabbit α-phosphorylated 4EBP1 (1:1000, 9451S, Cell Signaling Tech). The membranes were washed 3–5 times every 5 min with 1X TBS-T for a total of 30 min at room temperature with shaking. The membranes were then incubated with a secondary antibody conjugated to horseradish peroxidase HRP (1:1000 dilution) in 1X TBS-T with 5% BSA for 1 h at room temperature with shaking. Membranes were washed 3–5 times in 1X TBS-T at room temperature as previously described. Finally, SuperSignal West Femto Maximum Duration Substrate (ThermoFisher Scientific) was used to detection. Chemiluminescence was observed using an iBright 1500 system (Invitrogen) and the resulting file was exported for further analysis.

### Immunofluorescence and microscopy

Between 1.5 and 3.0 × 10^4^ U-2 OS cells/mL were seeded overnight on ethanol-sterilized 12-mm round glass coverslips (Azer Scientific 2000121) in 24-well tissue culture plates. The cells were then treated by replacing the media with media containing either NaAsO_2_ (200 μM) or DFO (100 μM, 500 μM, 1 mM), and incubated at 37 °C/5% CO_2_ for the indicated time. The cells were then washed with pre-warmed 1X PBS and fixed with 250 μL of 4% paraformaldehyde for 10 min at room temperature. After fixation, the cells were washed twice with 1X PBS, permeabilized with 0.1% Triton X-100 in 1X PBS for 5 min, and washed again with 1X PBS. Then, the cells were stained with specific primary antibodies (mouse α-G3BP1, 1:750, clone 2F3, ab56574, Abcam; rabbit α-PABP, 1:750, ab21060, Abcam; rabbit α-MEK1/2, 1:100, clone D1A5, 8727S, Cell Signaling Tech) that were diluted at the indicated concentration in 1X PBS. After overnight incubation in a humidified chamber, coverslips were transferred to a new 24-well plate, and washed three times for 10 min each with 0.1% Tween 20 in 1X PBS solution. The cells were then stained with 1:1000 goat α-rabbit or α-mouse secondary antibodies conjugated to FITC or Cy5 in 1X PBS for 1 h at room temperature. The coverslips were then washed three times with 0.1% Tween 20 in 1X PBS. Finally, coverslips were mounted onto slides using ProLong™ Glass Antifade Mountant with NucBlue™ Stain (Invitrogen) to stain the nuclei with Hoechst 33342.

Samples were imaged using a DeltaVision Elite microscope that was equipped with an Olympus UPlan-SApo 100X/1.40-NA oil objective and a PCO Edge sCMOS camera, and appropriate filters were used with the aid of SoftWoRx imaging software (University of Colorado-Boulder, BioFrontiers Advanced Light Microscopy Core).

### Quantification and statistics

ImageJ FiJi software was used to crop and quantify immunoblot and immunofluorescence images. Statistical significance was assessed by two-tailed Student’s t-tests. Significant differences are indicated by asterisks or different symbols above bars.

### Supplementary Information

Supplementary Figure S1.

Supplementary Information.

## Data Availability

The data presented in this study are openly available in Digital CSIC (https://digital.csic.es) at 10.20350/digitalCSIC/15724.
