# Supplementary material for: Regulation of translation in response to iron deficiency in human cells
Source: Sci Rep. 2024 Apr 11;14:8451. doi: 10.1038/s41598-024-59003-9 (PMC11009288; doi:10.1038/s41598-024-59003-9)
Supplement: Supplementary file 1 — Supplementary Figure S1. [file 41598_2024_59003_MOESM1_ESM.pdf]

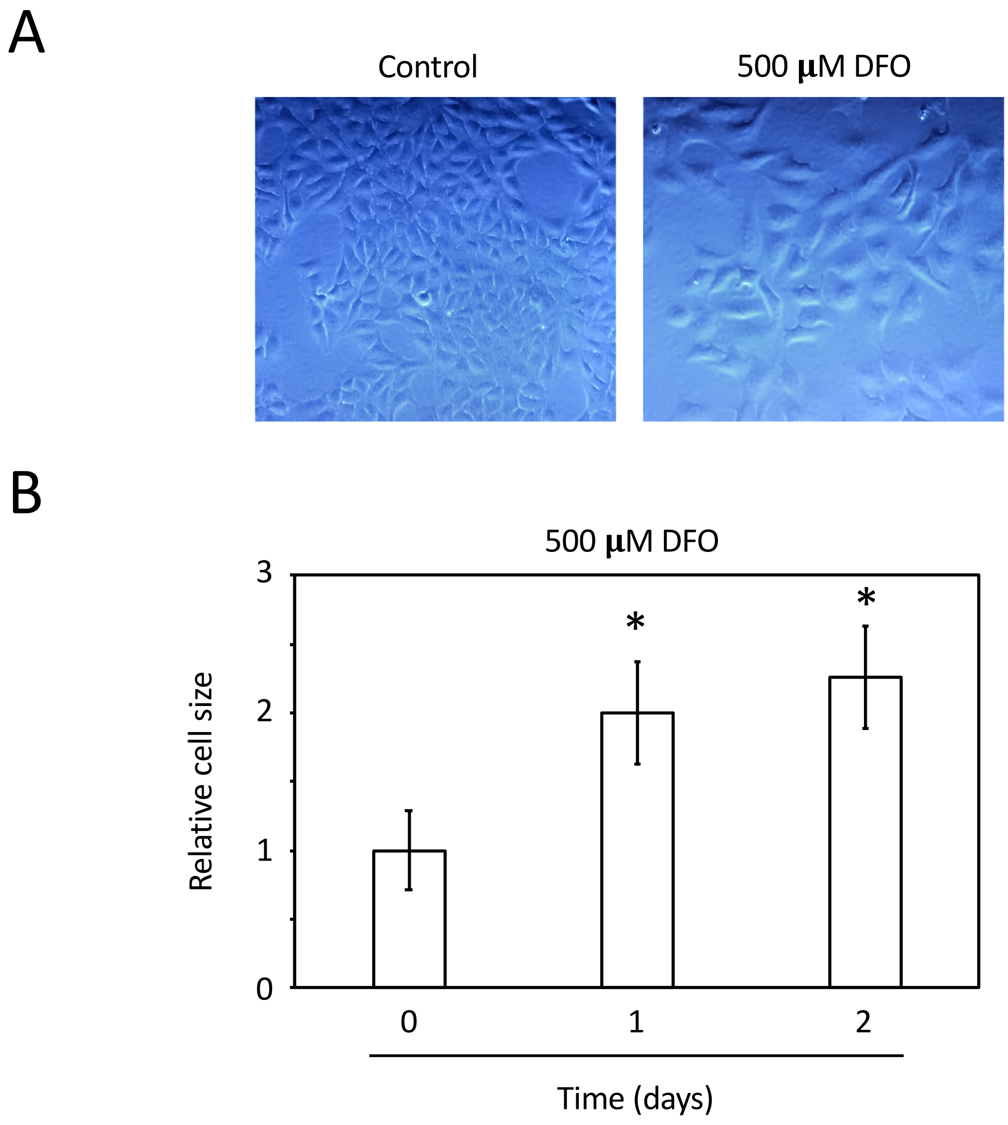

Figure S1: Cell size in iron-deficient conditions. U-2 OS cells were seeded and cultivated in the absence (control) or presence of 500 µM DFO for two days. (A) A representative photograph is shown. (B) The mean area without trypsin treatment (size) and standard deviation of 20 cells are represented. Values are relative to time zero. $^*p<0.05$.
