# Supplementary material for: Regulation of translation in response to iron deficiency in human cells
Source: Sci Rep. 2024 Apr 11;14:8451. doi: 10.1038/s41598-024-59003-9 (PMC11009288; doi:10.1038/s41598-024-59003-9)
Supplement: Supplementary file 2 — Supplementary Information. [file 41598_2024_59003_MOESM2_ESM.pptx]

## Slide 1
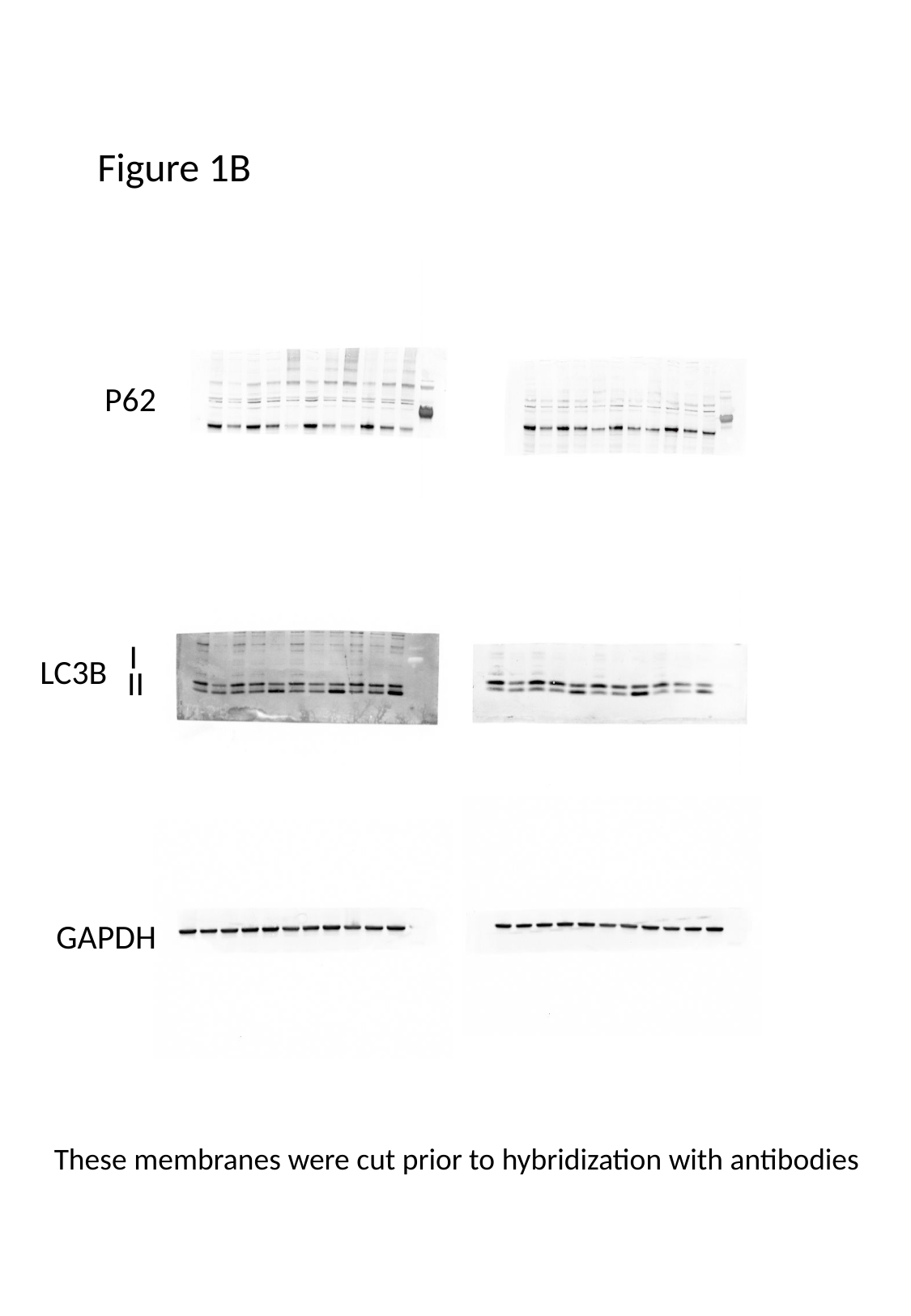

Figure 1B
P62
I
LC3B
II
GAPDH
These membranes were cut prior to hybridization with antibodies

## Slide 2
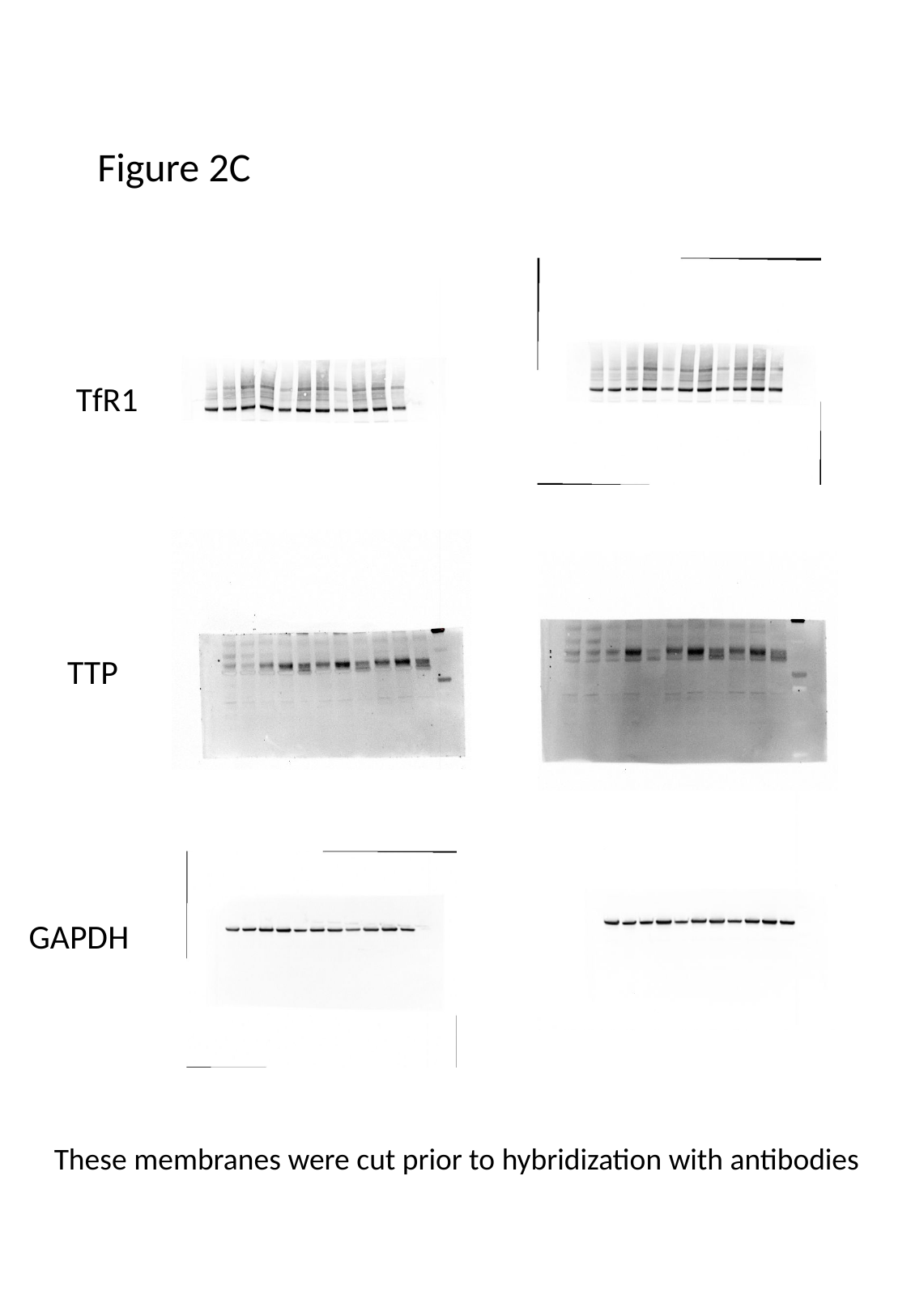

Figure 2C
TfR1
TTP
GAPDH
These membranes were cut prior to hybridization with antibodies

## Slide 3
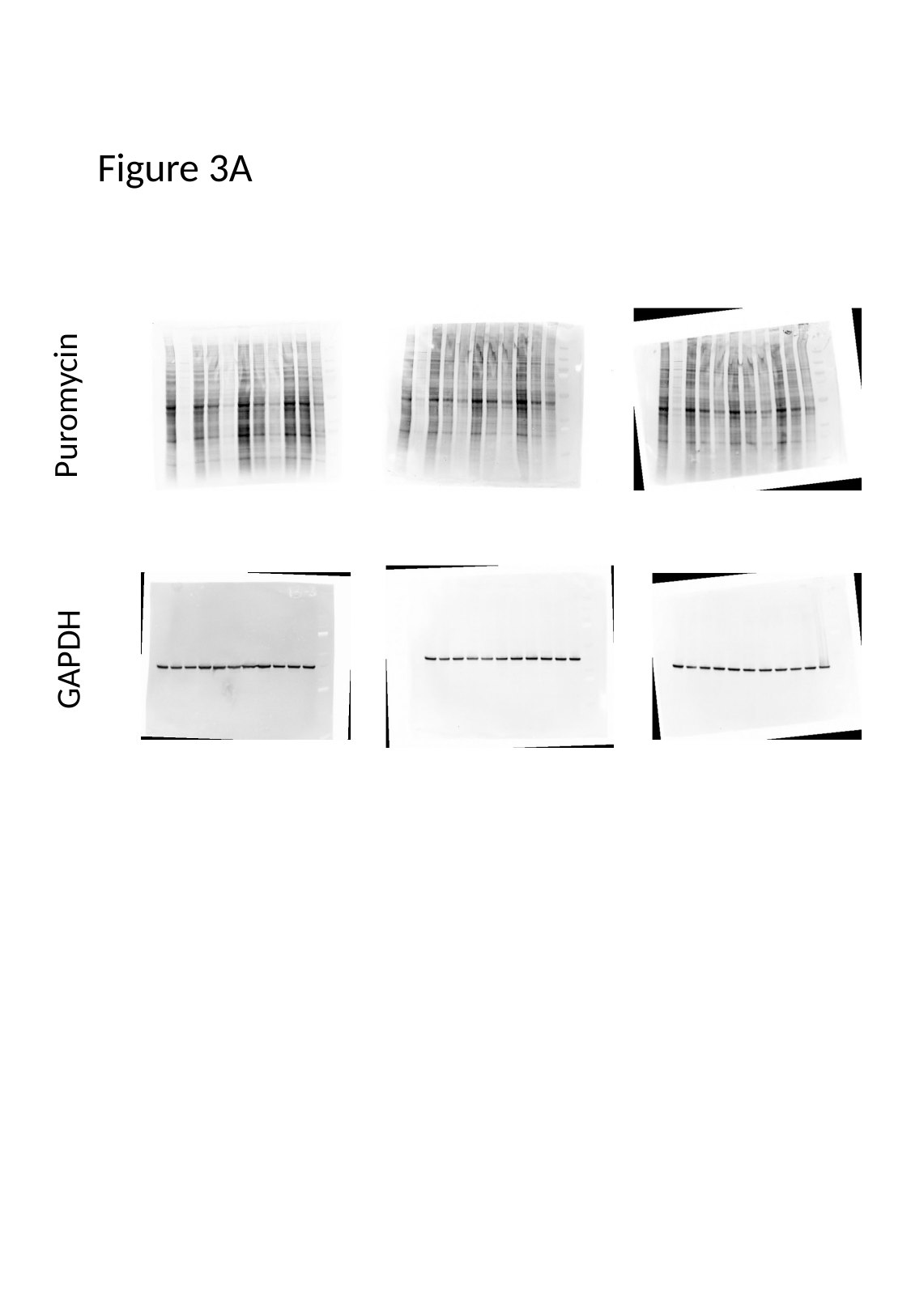

Figure 3A
Puromycin
GAPDH

## Slide 4
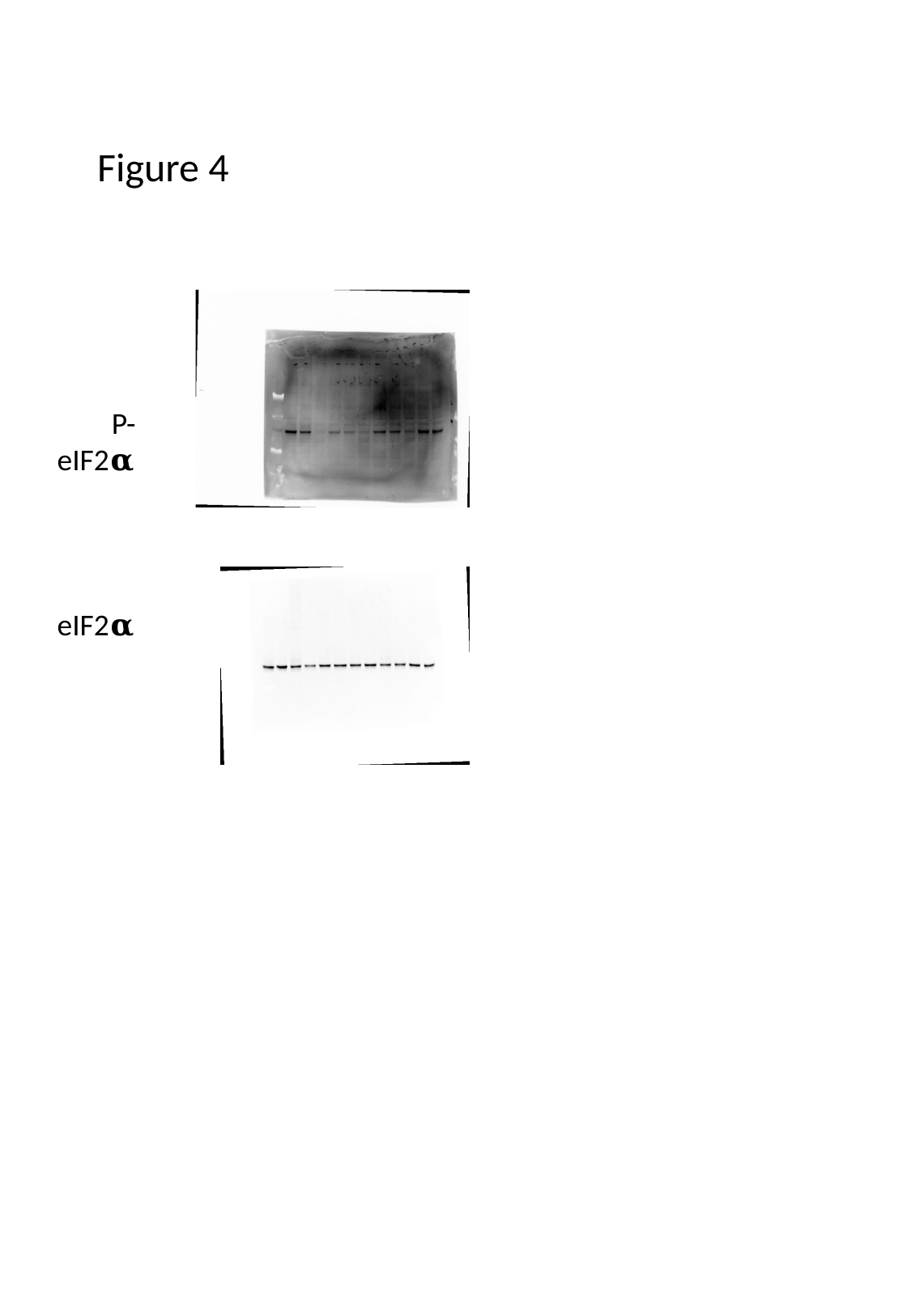

Figure 4
P-eIF2𝛂
eIF2𝛂

## Slide 5
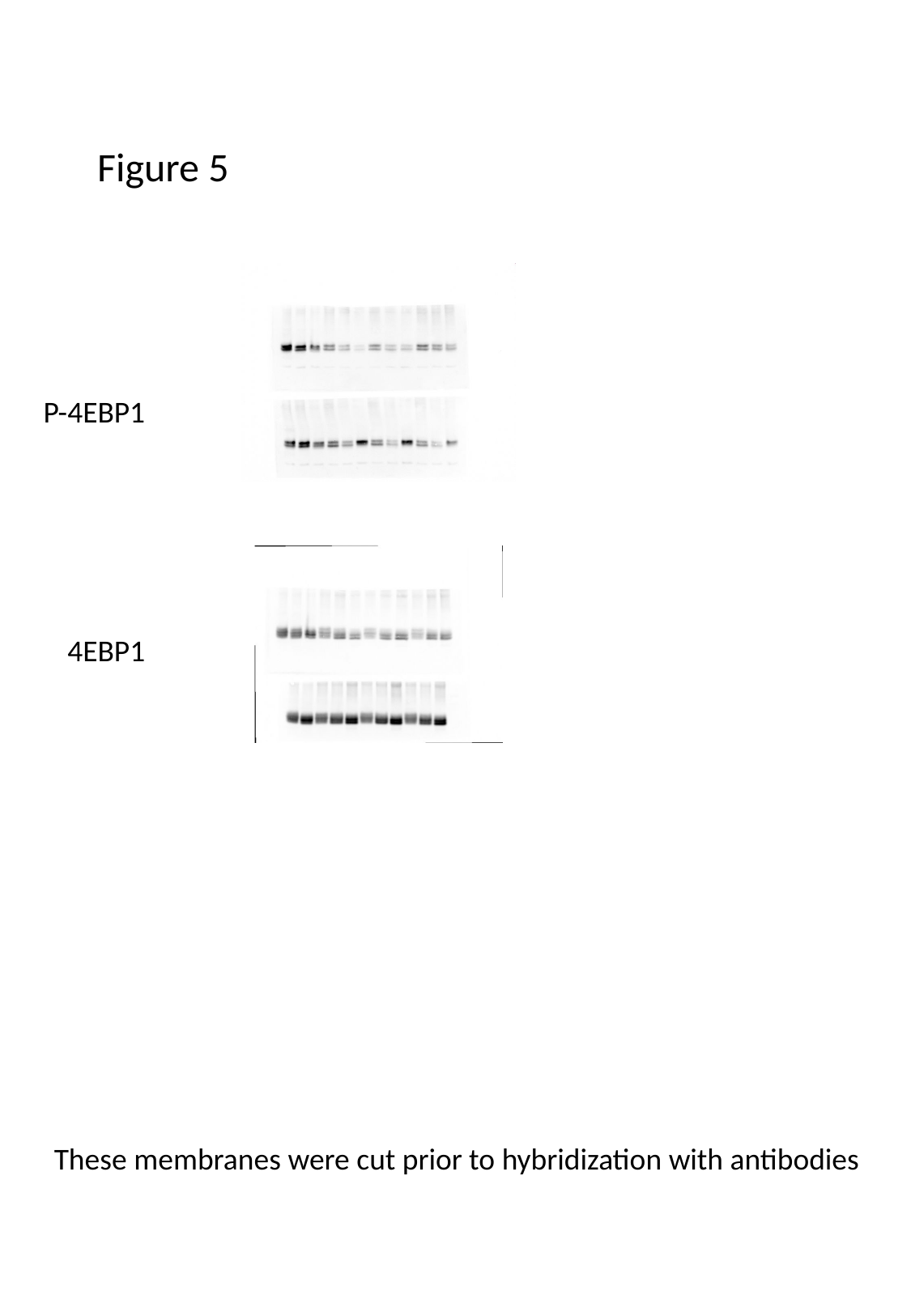

Figure 5
P-4EBP1
4EBP1
These membranes were cut prior to hybridization with antibodies
